# The Pharmacokinetics of Doxycycline in Channel Catfish (*Ictalurus punctatus*) Following Intravenous and Oral Administrations

**DOI:** 10.3389/fvets.2020.577234

**Published:** 2020-11-05

**Authors:** Ning Xu, Yu Fu, Bo Cheng, Yongtao Liu, Qiuhong Yang, Jing Dong, Yibin Yang, Shun Zhou, Yi Song, Xiaohui Ai

**Affiliations:** ^1^Yangtze River Fisheries Research Institute, Chinese Academy of Fishery Sciences, Wuhan, China; ^2^Hu Bei Province Engineering and Technology Research Center of Aquatic Product Quality and Safety, Wuhan, China; ^3^Key Laboratory of Control of Quality and Safety for Aquatic Products, Ministry of Agriculture and Rural Affairs, Beijing, China; ^4^Food Engineering College, Hunan University of Arts and Science, Changde, China; ^5^Aquatic Products Quality and Standards Research Center, Chinese Academy of Fishery Sciences, Beijing, China

**Keywords:** doxycycline, channel catfish, bioavailability, pharmacokinetics, non-compartmental model

## Abstract

The objective of this study was to investigate the bioavailability (BA) and pharmacokinetics (PK) of doxycycline (DC) in channel catfish (*Ictalurus punctatus*) following a single intravenous injection at 5 mg/kg and a single oral administration at 50 mg/kg at 24°C. The calculation of PK parameters was based on the software 3P97. The plasma samples were determined using ultra-performance liquid chromatography. Following oral administration, the multiple-peak phenomenon presented in concentration vs. time curve of DC at 2 h (107.01 mg/L), 8 h (55.07 mg/L), and 72 h (15.10 mg/L), respectively. The compartmental model cannot simulate the oral concentration vs. time profile beside a non-compartmental model. The calculated parameters of the elimination rate constant (λ_z_), the elimination half-life (t_1/2λ*z*_), and the area under the concentration vs. time curve (AUC_0*-*144_) were 0.037 1/h, 18.91 h, and 2255.45 μg.h/mL, respectively. After intravenous administration, the concentration vs. time profile of DC was best described by a two-compartmental open model without absorption. The parameters of the distribution rate constant (α), the distribution half-life (t_1/2α_), the elimination rate constant (β), the elimination half-life (t_1/2β_), the apparent distribution volume at steady state (V_ss_), the total clearance (Cl) and the area under the concentration vs. time curve (AUC_0-∞_) were 2.79 1/h, 0.25 h, 0.042 1/h, 16.51 h, 300.00 mL/kg, 14.00 mL/h/kg, and 364.99 μg.h/mL, respectively. For the calculation of BA values at the same condition, the data obtained from intravenous injection were also iterated based on a non-compartmental model, and the corresponding parameters of λ_z_, t_1/2λ*z*_, V_z_, Cl, and AUC_0*-*144_ were 0.019 1/h, 36.26 h, 480.00 mL/kg, 9.10 mL/h/kg, and 514.45 μg.h/mL, respectively. However, there was a considerable difference in the same parameter when calculated by compartmental and non-compartmental approaches. Finally, the medium BA value of DC was evaluated to be 43.84%. This study provides future studies with a framework for determining the BA of DC in the development of a new formulation and provides information on the appropriate use of DC in aquaculture.

## Introduction

Doxycycline (DC) is a semi-synthetic broad-spectrum antibiotic belonging to the tetracycline class. Compared with its analogs, DC possesses higher lipophilicity and permeability that results in extensive tissue disposition and long elimination half-life of the drug to efficiently kill pathogens (1). Based on these merits, DC has been approved for use in aquaculture against *Aeromonas hydrophila, Edwardsiella ictalurid*, and *Vibrio* in numerous countries, including China, Japan, and the Philippines (2). The label dose of DC is 20 mg/kg/day for 3–5 days for various fish species in China (2).

Channel catfish (*Ictalurus punctatus*) is a specific fresh-water fish species in China derived from the United States of America in 1987. As a food source, this fish species has many advantages. For instance, the muscles of channel catfish are free of intramuscular spines and contain tender and nutritious meat, which is typically suitable for consumption by children and the elderly. In response to the ever-increasing demands of consumers, the production of yellow catfish increased to >227,000 tons in 2017, meaning that this species occupies a primary position in China aquaculture (3). The intensified culture method has become increasingly popular to improve the production of channel catfish. However, this approach also results in serious problems in aquaculture: (a) water qualities progressively deteriorate due to the residue of formula feed; (b) fish are in poor health due to high culture density and excess nutrient intake; (c) fish diseases can easily develop, and occur in a restricted culturing environment in response to weather and temperature changes, as well as other stress factors. These diseases can lead to a high mortality rate, which also results in economic loss. *Edwardsiellosis* is a serious disease caused by *E. ictalurid*, a particularly important pathogen in channel catfish. This organism can result in deep tissue infection reaching the brain. Consequently, it is difficult to cure this disease due to the low concentration of the drug that reaches the infection site (4). Hence, we need to develop a drug with good permeability that can pass through the blood-brain barrier. DC is a good candidate for the treatment of this disease because of its aforementioned merits but an investigation of the related pharmacokinetics (PK) of DC has yet to be conducted in channel catfish.

It is well-established that bioavailability (BA) is an important parameter related to the speed and degree of drug absorption into the animal body. It describes the rate and extent to which a substance or its active moiety is delivered from a pharmaceutical form via absorption by gastrointestinal tract or other administered site by non-vascular dosing (5). In this regard, the study of BA is important in developing a pharmaceutical formulation. An increase in drug BA suggests that more of the drug has entered the blood circulation, enhancing the therapeutic effect. Consequently, in the development of drug preparations, their BA values are often compared to obtain the optimal formulation. However, the study of BA in aquatic animals has not received considerable attention from researchers.

Numerous PK studies of DC have been conducted in chickens (6), ducks (7), pigs (8), sheep (9), and goats (10). Few studies have been implemented in fish, such as tilapia (11) and grass carp (12, 13). However, there is limited available information on the BA of DC in channel catfish. Therefore, the present study aimed to investigate the BA and PK characteristics in channel catfish, which will provide fundamental data for the development of a new formulation of DC in the future and efficient use of this agent in channel catfish.

## Materials and Methods

### Chemicals and Reagents

The analytical standard of DC (>98.0% purity) was obtained from Dr. Ehrenstorfer GmbH. (Augsburg, Germany). The purity of DC powder (active pharmaceutical ingredient) used for drug administration was >98.0%. The DC was provided by Zhongbo Aquaculture Biotechnology Co. Ltd. (Wuhan, China). The liquid solvents of acetonitrile, methanol, water, and formic acid were bought from J–T Baker (Philipsburg, NJ, United States) and Thermo Fisher (Waltham, MA, United States). Sodium dihydrogen phosphate, ethylenediaminetetraacetic acid disodium (EDTA-Na_2_), and citric acid monohydrate were purchased from Shanghai Guoyao Company (Shanghai, China). The 1.5 mL vials, 0.22-μm politetrafluoroetileno membranes, and centrifugal tubes were provided by Shanghai CNW Technologies (Shanghai, China).

### Animals and Management

Two hundred channel catfish of both sexes (150.3 ± 10.2 g) were purchased from the culture facility of the Yangtze River Fisheries Research Institute (Wuhan, China) and held in water tanks (10 fish per tank; the volume of each tank: 480 L) with flowing well water (26 L/min). Prior to the formal experiment, channel catfish were acclimatized for 2 weeks fed with no-antibiotic formula rations containing 30.81% crude proteins, 5.27% crude fat, and 8.88% ash (14). These feeds were produced by the Nutritional Research Group at the Yangtze River Fisheries Research Institute, Chinese Academy of Fishery Sciences, Wuhan, China. Water quality parameters were measured daily and maintained at an appropriate level (pH at 7.4 ± 0.2; dissolved oxygen levels at 6.6–7.5 mg/L; total ammonia nitrogen levels ≤ 0.75 mg/L; nitrite nitrogen levels <0.08 mg/L; and water temperature:24.0 ± 0.3 °C controlled by aquarium heater and air-conditioner). To get a negative control and blank sample for ultra-performance liquid chromatography (UPLC) analysis of DC, the blank plasma was collected from fish fed with no-antibiotics feed. Animal experimental procedures were approved by the Fish Ethics Committee of the Yangtze River Fisheries Research Institute, Chinese Academy of Fishery Sciences, Wuhan, China.

### Oral Administration, Intravenous Administration, and Sampling

The method for the preparation of the DC solution for oral administration and in the experiment involved oral gavage and sampling, as previously reported (15, 16). The active pharmaceutical ingredient of DC was used to prepare the solution at a final concentration of 20 mg/kg. The DC solution was given to each fish at a dose of 50 mg/kg using a hard-plastic tube attached to a 1 mL micro-injector. There was no use of any anesthetic during the experiment to avoid an effect on PK properties (17, 18). After oral gavage, if the fish regurgitated the given DC solution, the fish was removed from the tank and replaced by another. The sampling time points were at 0.083, 0.17, 0.5, 1, 2, 4, 6, 8, 16, 24, 48, 96, and 144 h after oral dosing. At each time point, ~2 mL blood was drawn from the caudal vessels of each fish (six fish in total). Subsequently, plasma samples were obtained by centrifugation of corresponding blood samples at 1,500 g for 5 min, and stored at −20°C up to analysis.

The DC solution for intravenous administration was made by dissolving DC into physiological sodium water (0.9%) at a final concentration of 5 mg/L. To guarantee the solubility of DC in water (clear and not turbid), an appropriate amount of hydrochloric acid was added to the solution. The detailed methods of intravenous treatment and sampling were in line with previous reports (19, 20). Firstly, a 22 G needle with a 1-mL syringe was pre-inserted into the target area to confirm the correct position of the caudal vein by aspirating blood into the syringe. Next, the DC solution was injected into the caudal vein at a dose of 5 mg/kg without using any anesthetic. Following intravenous administration, in the presence of continuous bleeding, the fish was removed from the tank and replaced by untreated fish. Subsequently, blood sampling and processing were performed (as described above) for oral gavage.

### Sample Preparation and HPLC Analysis

The sample preparation and conditions of the UPLC analysis were based on the methods of a previous report (12). In brief, 1 mL of plasma samples (thawed at room temperature) was separately pipetted into polypropylene tubes (10 mL). Next, 5 mL of EDTA-Mcllvaine buffer (0.04 mol/L sodium dihydrogen phosphate, 0.06 mol/L citric acid monohydrate, and 0.1 mol/L EDTA-Na_2_, pH = 4.0) was added to each sample. The mixture was shaken for 2 min, and centrifuged for 5 min at 5,000 g at 4°C. Each sample was extracted twice through this approach. The resulting supernatants were pooled into a new tube and transferred to a polymeric SPE cartridge prepared beforehand with 3 mL of methanol and 3 mL of pure water. Following the percolation of the whole solution, the columns were washed with 3 mL of water. The DC was eluted with 5 mL of methanol. The extracts were evaporated using nitrogen at 30°C until completely dry. The dry residues were reconstituted using 1 mL of 5% methanol in water (0.1% formic acid) and filtered through 0.22 μm filters. The samples (10 μL per sample) were used for UPLC analysis.

A Waters ACQUITY™ UPLC (Milford, MA, United States) equipped with a binary solvent manager with a binary solvent pump, a sampler manager with an autosampler, and an ultraviolet detector were used to analyze plasma samples. An ACQUITY UPLC BEH C18 column (1.7 μm, 2.1 × 100 mm) was used to separate the chemical. The column temperature was set at 30°C. The flow rate was 0.35 mL/min with a mobile phase including pure water (0.1% formic acid) and acetonitrile (0.1% formic acid). The proportion of the aqueous phase and the organic phase was 70: 30. The detection wavelength was set at 350 nm.

### Validation Procedures

The validation procedures of the UPLC analytical method for DC in fish plasma was based on specific indicators involving the limit of detection (LOD), the limit of quantification (LOQ), selectivity, linearity, precision, and accuracy (21). The LOD was established based on the concentration that produced the area of the signal 3 times that of the baseline noise; the LOQ was determined as the concentration that produced the ratio of the area of the signal to the baseline noise of 10. The selectivity of the method was assessed by detecting blank plasma samples and the samples fortified with DC standard solution. Through a comparison of chromatograms, we sought to determine whether the DC peak was intact and if there was interference in the chromatogram. In terms of interference in the signal plot, the extraction method and the separation method for DC on UPLC were optimized. In this study, the matrix-matched curves of DC in plasma were used to quantify the DC concentration by analyzing samples spiked with different concentrations of 0.05, 0.1, 0.5, 2.0, 5.0, and 20.0 μg/mL to establish the calibration profile. The calibration curve was constructed by regressing the area of the chromatographic peak with the concentration of DC, and the correlation coefficient was also calculated. The accuracy of the method was evaluated by the recovery rate, which was determined by analyzing blank samples fortified in five replicates at levels of 0.05, 0.5, and 5.0 μg of DC per mL. For the assessment of method precision, the repeatability values (intra-day precision) were analyzed as the coefficient of variation of the measured concentrations of DC by analyzing samples fortified with a standard of the analyst at three levels (0.05, 0.5, and 5.0 μg/mL) on the same day with the same instrument and by the same operator. The reproducibility (intermediate precision) results were obtained by determining samples spiked with target compounds using the identical method on three separate days with the same instrument and by the same operator.

### Pharmacokinetic Analysis

The software 3P97 (Math-pharmacology Committee, Chinese Academy of Pharmacology, Beijing, China) was used to analyze DC plasma vs. time profiles with non-compartmental and two-compartmental approaches. For the selection of a suitable PK model, the study conducted a visual examination of the concentration-time curve, and calculation of the value of Akaike's Information Criterion (22). The following PK parameters were estimated: λ_z_ (elimination rate constant of non-compartmental model), t_1/2λ*z*_ (elimination half-life of non-compartmental model), T_max_ (time to peak concentration), C_max_ (maximum concentration), AUC_0*-*144_ (area under the concentration vs. time curve from 0 to 144 h), AUC_0*-*144_/D (area under the concentration vs. time curve from 0 to 144 h calibrated by dose), AUC_0-∞_ (area under the concentration vs. time curve extrapolated to infinity), V_z_ (apparent distribution volume at elimination phase of non-compartmental model), and Cl (systemic total body clearance); A (zero-time blood drug concentration intercept of distribution phase); B (zero-time blood drug concentration intercept of elimination phase); α (distribution rate constant of two-compartmental model), β (elimination rate constant of two-compartmental mode), t_1/2α_ (distribution half-life of two-compartmental mode), t_1/2β_ (elimination half-life of two-compartmental mode), K_10_ (drug elimination rate constant from central compartment), K_12_ (first-order transport rate constant from central compartment to peripheral compartment), K_21_ (first-order transport rate constant from peripheral compartment to central compartment), and V_ss_ (apparent distribution volume at steady state of two-compartmental model).

The ratio of the calculated area under the concentration-time curve after oral administration to intravenous injection was used to estimate the oral BA value using the equation described below:

$$\begin{array}{r} \text{F}=\frac{\left(\text{AUC oral administration}\right)\times \left(\text{dose intravenous injection}\right)}{\left(\text{AUC intravenous injection}\right)\times \left(\text{dose oralãdministration}\right)}\times 100\text{\%} \end{array}$$

## Results

### UPLC Methodology

The UPLC method of DC was validated by specific indices of LOD, LOQ, selectivity, linearity, recovery, and precision. There were no interference peaks observed in the chromatograms of spiked samples. The LOD and LOQ of DC were determined as 25 and 50 μg/L in spiked plasma samples, respectively. The matrix-match calibration curve was established across concentrations of 0.05–20.0 μg/mL, with good linearity by the coefficient of correlation (*R*^2^ = 0.999). During UPLC determinations, if the concentration in samples in the initial analysis was more than the upper LOQ, the remaining samples were repeatedly measured after dilution with the relevant blank plasma. The mean recovery rates of DC ranged from 69.8 to 83.2% in plasma, as listed in Table 1. Their percentage of relative standard deviations for inter-day and intra-day precision were <10% displayed (Table 1).

**Table 1:** Accuracy and precision of the UPLC method for doxycycline in fortified plasma of channel catfish (*Ictalurus punctatus*) (*n* = 5).

**Fortified concentration**	**Recovery (%)**	**Within-day**	**Between-day**
**(μg/mL)**		**RSD (%)**	**RSD (%)**
0.05	83.2	4.5	5.9
0.5	78.3	3.7	5.3
5.0	69.8	4.0	6.1

### Pharmacokinetic Properties of DC in Channel Catfish

**Figure 1:**
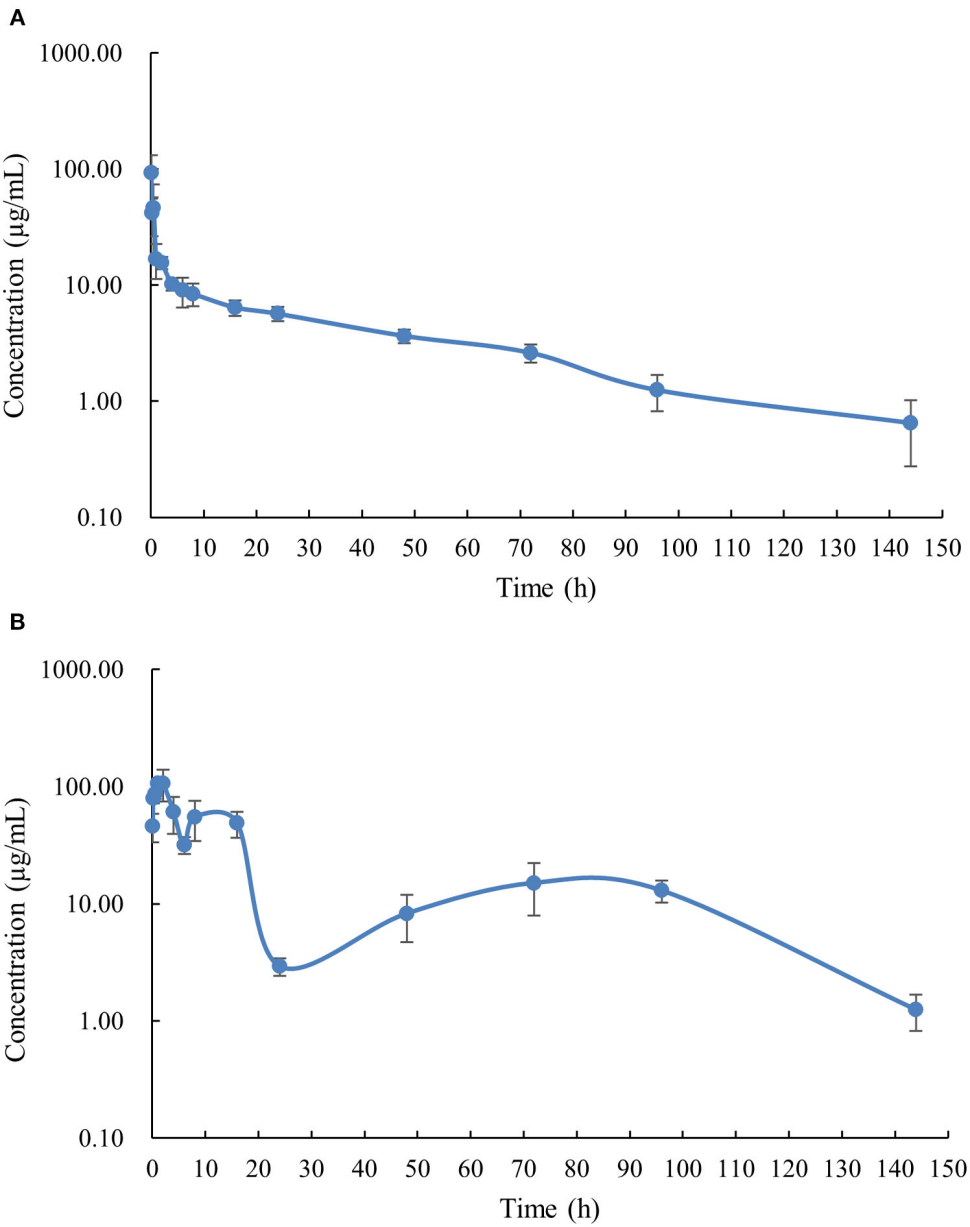
The plasma concentration-time curve with logarithmic scales of doxycycline in channel catfish (*Ictalurus punctatus*) after intravenous administration at a dose of 5 mg/kg **(A)** and oral administration at a dose of 50 mg/kg **(B)**.

**Figure 2:**
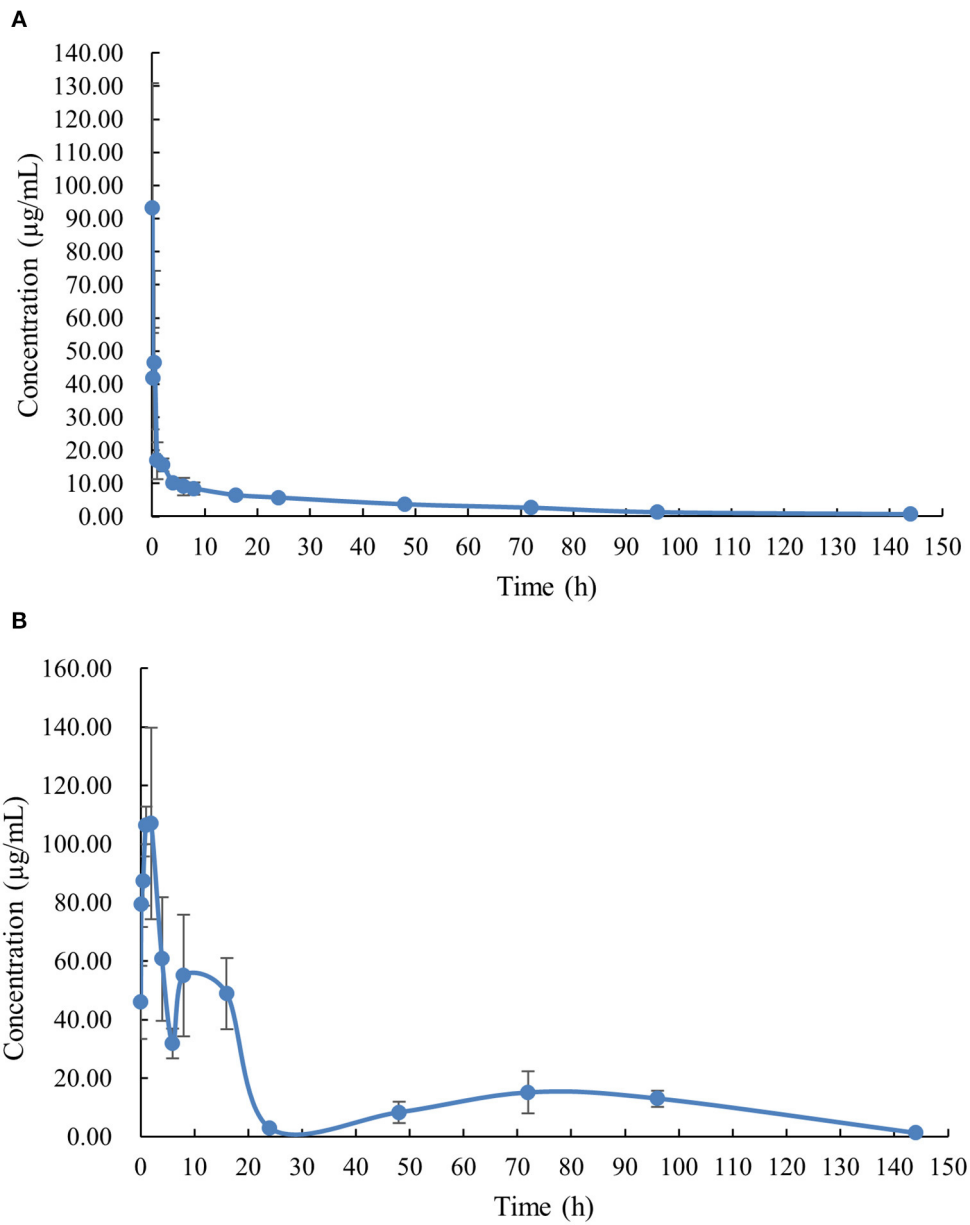
The plasma concentration-time curve with normal scales of doxycycline in channel catfish (*Ictalurus punctatus*) after intravenous administration at a dose of 5 mg/kg **(A)** and oral administration at a dose of 50 mg/kg **(B)**.

The PK profile of DC in channel catfish following a single oral gavage at 50 mg/kg and a single intravenous treatment at 5 mg/kg are shown in Figures 1, 2. After oral gavage, the results display multiple-peak phenomenon in the concentration vs. time curve. The first peak concentration (107.01 μg/mL) was found at 2 h after oral dosing. Subsequently, the concentration started to decline and reached the second peak concentration (55.07 μg/mL) at 8 h. Next, the concentration decreased again and reached the third peak concentration (15.10 μg/mL) at 72 h. The concentration was then gradually decreased. Following intravenous injection, the results exhibited the highest DC concentration of 93.14 μg/mL at the first sampling point, declined to 41.70 μg/mL at 0.167 h, increased to 46.46 μg/mL at 0.5 h, and progressively decreased thereafter.

The PK parameters were listed in Tables 2, 3. The concentration vs. time profile after oral gavage cannot be iterated by a compartmental model due to the irregular multiple-peak phenomenon; it was thus, only simulated by a non-compartmental model. The main parameters of T_max_, C_max_, λ_z_, t_1/2λ*z*_, and AUC_0*-*144_ were 2 h, 107.01 μg/kg, 0.037 1/h, 18.91 h, and 2255.45 μg.h/mL, respectively. The concentration vs. time profile following intravenous injection was best described by a two-compartmental model. The main parameter of α, t_1/2α_, β, t_1/2β_, V_ss_, Cl, and AUC_0-∞_ were 2.79 1/h, 0.25 h, 0.042 1/h, 16.51 h, 300.00 mL/kg, 14.00 mL/h/kg and 364.99 μg.h/mL, respectively. To estimate the oral BA value using the same iteration method, the PK parameters receiving intravenous treatment were calculated by a non-compartmental model. The main parameters λ_z_, t_1/2λ*z*_, V_z_, Cl and AUC_0*-*144_ were 0.019 1/h, 36.26 h, 0.48 L/kg, 0.0091 mL/h/g, and 514.45 μg.h/mL, respectively. Finally, the oral BA value was estimated at 43.84%.

**Table 2:** Pharmacokinetic parameters of doxycycline in channel catfish (*Ictalurus punctatus*) following intravenous administration at a dose of 5 mg/kg calculated by a two compartmental model (*n* = 6).

**Parameters**	**Unit**	**Values**
A	μg/mL	81.28
B	μg/mL	14.1
α	1/h	2.79
β	1/h	0.042
t_1/2α_	h	0.25
t_1/2β_	h	16.51
C_max_	μg/mL	95.37
K_10_	1/h	0.26
K_12_	1/h	2.12
K_21_	1/h	0.45
V_ss_	mL/kg	300.00
Cl	mL/h/kg	14.00
AUC_0-∞_	μg*h/mL	364.99

*A: zero-time blood drug concentration intercept of distribution phase, B: zero-time blood drug concentration intercept of elimination phase, α: distribution rate constant of two-compartmental mode, β: elimination rate constant of two-compartmental mode, t_1/2α_: distribution half-life of two-compartmental mode, t_1/2β_: elimination half-life of two-compartmental mode, C_max_: the maximum concentration, K_10_: drug elimination rate constant from the central compartment, K_12_: first-order transport rate constant from central compartment to peripheral compartment, K_21_: first-order transport rate constant from peripheral compartment to central compartment, V_ss_: apparent distribution volume at steady state of two-compartmental mode, Cl: systemic total body clearance, AUC_0-∞_: area under the concentration vs. time curve extrapolated to infinity*.

**Table 3:** Pharmacokinetic parameters of doxycycline in channel catfish (*Ictalurus punctatus*) after intravenous administration at a dose of 5 mg/kg and oral gavage at a dose of 50 mg/kg calculated by a non-compartmental model (*n* = 6).

**Parameters**	**Unit**	**iv**	**p.o**.
λ_z_	1/h	0.019	0.037
t_1/2λ_	h	36.26	18.91
T_max_	h	0.083	2.00
C_max_	μg/mL	93.14	107.01
AUC_0*-*144_	μg*h/mL	5144.50*	2255.45
AUC_0*-*144_/D	kg.h/mL	0.10	0.045
V_z_	mL/kg	480.00	NA
Cl	mL/h/kg	9.10	NA
F	%	NA	43.84

i.v.: intravenous, p.o.: per os, λ_z_: elimination rate constant of non-compartment mode, t_1/2λ_: elimination half-life of non-compartment mode, T_max_: the time to peak concentration, C_max_: the maximum concentration, AUC_0-144_: area under concentration vs. time curve from 0 to 144 h, AUC_0-144_/D : area under concentration vs. time curve from 0 to 144 h calibrated by dose; V_z_: apparent distribution volume at the elimination phase of non-compartment mode, Cl: systemic total body clearance, NA: not applicable, *: the iv AUC after normalized by oral dose.

## Discussion

This study examined the PK characteristics of DC in channel catfish following oral and intravenous administration. The main findings were: (a) the multiple-peak phenomenon was presented in the drug concentration vs. time profile after oral dosing; (b) the AUC of oral administration was markedly greater than that of intravenous injection, calculated by a non-compartmental approach; (c) a medium BA of DC (43.84%) was found in channel catfish. The concentration-time profile of DC exhibited multiple-peak phenomenon after oral administration; thus, it could not be described by the classical approach of the compartmental model, except for the non-compartmental model. In previous studies, the concentration-time curve of DC also displayed multiple-peak phenomenon. There were two peaks displayed at 2 and 24 h after oral administration in tilapia (*Oreochromis aureus* × *Oreochromis niloticus*) at a single dose of 20 mg/kg at 24°C (11). In the PK study of DC in grass carp (*Ctenopharyngodon idella*) at 18 and 24°C, more peak concentrations were displayed in the concentration-time curves (12). At 18°C, five peak concentrations occurred at 1, 4, 12, 72, and 120 h, respectively, after a single oral dose of 20 mg/kg (12). At 24°C, three peak concentrations were exhibited at 1, 4, and 72 h, respectively, after oral dosing of 20 mg/kg. In the present study, there were three peaks exhibited in the plasma concentration-time curve at 2, 8, and 72 h, respectively. Moreover, multiple-peak phenomena were also found in PK studies of ducks (7), pigs (23), and humans (24). The observed fluctuations in DC concentrations could be partly due to the impact of enterohepatic recycling. DC has a high affinity for bile and might form stable complexes with bile that re-enter the intestine via the biliary excretion (24, 25). The complexes might also be degraded at the role of digestion liquid and microflora during peristalsis, leading them to discontinuously reabsorb because of pH changes in the digestion liquid, subsequently causing the occurrence of a secondary peak in plasma (23). Hence, plasma data were not iterated by the classical compartmental PK model. However, they can be iterated by a non-compartmental model or a physiological model involving elements of discontinuous cyclic transfer that may be more suitable for simulating plasma concentration-time data.

After a single oral dose of 50 mg/kg at 24°C in this study, the observed AUC was greater than that following intravenous administration at a dose of 5 mg/kg at the same temperature. This difference may be explained by enterohepatic circulation, causing reabsorption of oral DC in the intestine, as indicated by the results of this study, which showed different concentration peaks, which presented three times in the plasma concentration-time curve and possibly leading to the slow elimination of DC *in vivo*. Hence, the oral area shaped from the concentration of DC with time was also enlarged. However, following intravenous dosing, no conspicuous multiple-peak phenomenon was presented in the concentration-time profile accompanied by the lack of an apparent increase in AUC. This may be due to the entry of most of the drug into the blood circulation system. Therefore, we speculated that a small amount of DC entered the liver and a limited amount of DC was excreted from the liver to bile. Thus, a concentration peak was not observed. A second reason was the relatively high dose used in oral administration, which was ten times higher than that of intravenous injection. Consequently, the C_max_ and the terminal elimination time increased. Therefore, it is not unexpected that the AUC was increased in oral gavage. In addition, the AUC value was higher than that in grass carp at the same temperature, suggesting that the persistent time in the body was longer for channel catfish than that in grass carp (12). Subsequently, the value of t_1/2λ*z*_ was 18.91 h, which is close to that noted in grass carp (20.10 h) but shorter than that for tilapia (77.20 h) at the same temperature (11, 12). In summary, these differences might be due to the different dosages used in these studies and the discrepancies in the disposition of DC in different fish species. Moreover, in a study of the pharmacokinetic-pharmacodynamic (PKPD) model of DC against *A. hydrophilia*, part of PK data of DC was reported in channel catfish following oral gavage at a dose of 20 mg/kg at 28°C (26). A multiple-peak phenomenon was not found in that study. A two-compartmental model with first-order absorption was employed to simulate the concentration-time profile. These results are different from those of the current study, but the reasons for this are unknown. The T_max_ value (2.57 h) was similar to that of this study (2.0 h). However, the C_max_ value of 1.72 μg/mL was considerably less than that of this study (107.01 μg/mL), and the t_1/2β_ value of 38.63 h was approximately two-fold of the current study. These marked discrepancies might be due to the different experimental doses and temperatures between the two studies.

Different PK values were obtained for the same data group using different iteration approaches, the compartmental model and the non-compartmental model. Using the established strategy of a two-compartmental model to iterate data following intravenous injection, the calculated values of t_1/2β_, V_ss_, CL, and AUC_0-∞_ were 16.51 h, 300.00 mL/kg, 14.00 mL/h/kg, and 364.99 μg.h/mL, respectively. These values were different to the corresponding values (36.26 h, 480.00 mL/kg, 9.10 mL/h/kg, and 514.45 μg.h/mL) calculated using a non-compartmental model. Therefore, we estimated different values of BA (61.79 vs. 43.84%) from the same data group. These discrepancies may be due to different assumptions between the compartmental and non-compartmental models. Compartmental methods consider that the body consists of a finite number of interconnected, well-mixed, and kinetically homogeneous compartments. Hence, the PK scientist can make certain assumptions and establish a model using non-linear regression analysis to simulate the PK profile of the drug. Since the assumptions used to develop PK models may be somewhat different, the results of the analysis may vary (27, 28). In contrast, non-compartmental analysis (NCA) is model-independent, meaning it does not rely on assumptions regarding body compartments and tends to provide more analyst-to-analyst consistency. Moreover, NCA depends almost exclusively upon the algebraic equation to estimate PK parameters, rendering the analysis less complex than that of compartmental methods (27, 28). Therefore, NCAs are often faster and more cost-efficient, especially vs. complex compartmental analysis.

The BA value is an important indicator in investigating the PK properties of drugs in animals (29). A BA value of 35.77% has been reported in sheep receiving oral administration at a dose of 20 mg/kg (9). It was estimated that the BA values in turkeys exposed DC via oral dosing ranged from 40.0 to 83.7% (30). In Muscovy ducks, after oral administration at a dose of 20 mg/kg, the BA values of DC were observed to range from 39.13 to 70.71% (7). A greater BA value was determined at 545.00% in goats receiving a long-acting parenteral formulation of DC hyclate at a dose of 10 mg/kg (10). These reports indicate that long-acting formulation generally possesses prolonged absorption, possibly resulting in flip-flop kinetics. Therefore, the reason may be that the slope used for extrapolation to infinity was not representative of elimination and the extrapolated AUC was larger than it would be if concentration were measured for a longer period.

Overall, the variability of the BA value of DC is diverse in response to variations in animal species, formulations, and other impact factors. To achieve better efficacy in the development of drug formulations, it is necessary to conduct BA studies for different formulations. Another study reported the PKs of two oral formulations of DC (Providox® (DC hydrochloride power, 200 mg/g, Amman, Jordan) and Doxyvet 0-50S® (DC hydrochloride power, 500 mg/g, Arendonk, Belgium)) in chickens. The relative BA value of Providox® compared with Doxyvet 0-50S® was 108.24%, suggesting the two formulations possessed comparable efficacy (31). Another report also estimated the PKs of Doxysol® (DC hydrochloride power, 200 mg/g, Ascor Chimici, Italy) and Doxymed® (DC hydrochloride power, 200 mg/g, Amman, Jordan) in different preparations of DC provided by a commercial company in broilers (32). The mean systemic BA values of DC in Doxycol® and Doxymed® following oral dosing were 92.57 and 88.21%, as well as the AUC_test_/AUC_reference_ ratio was 90%, indicating that Doxysol® was bioequivalent to Doxymed®. However, few studies have calculated BA values for aquatic drugs in fish. Possible reasons may be: (a) the experimental method of intravenous injection is difficult for general scientists that limited the BA determination; (b) the studies on the dosage form in fish have not received adequate attention from researchers. To improve this situation, we carried out several BA studies of aquatic drugs in fish, such as mebendazole (20), flumequine (19), and praziquantel (16). The BA value of minocycline has also been reported in the crucian carp (*Carassius auraus*) (33). In the present study, a medium BA value of DC was determined as 43.83% that was about two-fold that in tilapia (23.41%) at an oral dose of 20 mg/kg at 24°C. Moreover, a value of 66.08% was found in grass carp at an oral dose of 20 mg/kg at 24°C that was even more than that in channel catfish and tilapia, suggesting that more DC can enter the body of grass carp to reach a higher concentration in plasma and maintained for a longer efficient time (12). The given dose and time intervals in grass carp should be lower or shorter, respectively, than those determined in channel catfish and tilapia. The different BA values of DC presented in different fish species is partly attributable to different dosages, different environmental factors, and disparate mechanisms of absorption and the metabolism of DC in different fish species.

It is well-established that PK studies can establish the therapeutic dosage regimen for a target disease. In fish, the minimum inhibitory concentrations (MICs) of DC were 2 μg/mL against *A. hydrophila* (26) and 0.5 μg/mL against *Streptococcus spp*. (11), respectively. The PKPD indices of C_max_/MIC, AUC/MIC, and T>MIC are often used as the predictors of therapeutic efficacy (34, 35). Generally, DC is considered as a time-dependent drug, thus T>MIC is a suitable indicator for determining the dosage and time interval of drug administration (36). However, it has been reported that the DC presented a time-dependent profile at low concentration but a concentration-dependent profile at high concentration against *Escherichia coli, Staphylococcus aureus, Pasteurella multocida*, and *Streptococcus pneumoniae* (37). Similar results were also shown in a PKPD study of gentamicin that displayed a time-dependent killing kinetic opposing *Staphylococcus aureus* and a concentration-dependent killing kinetic resisting *Pseudomonas aeruginosa* (38). The reason for this difference may be due to the different target microorganism (37, 38). Furthermore, another previous study of the PKPD of DC against *A. hydrophila* did not select an optimal PKPD indicator (26). The PKPD study of DC was not carried out against pathogens in this study, and we temporarily considered DC as a time-dependent drug. According to this assumption, T>MIC was 96 h for *A. hydrophila* after a single oral dose of 50 mg/kg at 24°C. For the susceptible bacteria of *Streptococcus spp*., T>MIC was 144 h after a single oral dose of 50 mg/kg at 24°C. Although the dosage of 50 mg/kg has not been used in aquaculture, it will provide useful information for improving the dosage regime for anti-drug resistance.

In the present study, the blood samples were obtained from six individual fish at each sampling time point. Individual fish may exhibit some differences in physiology, physiochemistry, and biochemistry that will influence drug disposition *in vivo*. Although no specific studies report on the PK differences in individual fish, numerous reports involving humans and land animals have provided relevant information (39–43). Individual variabilities were mainly related to the absorption of the drug, metabolism (genetic factors and environmental factors), the drug levels in tissues, and drug-protein binding, etc. (44). These factors have indicated disparate PK behaviors among individuals of land animals and the same factors in fish may also similarly impact PK properties. Therefore, the current method of sampling from multiple fish at each time point cannot distinguish interindividual variation. Although we were aware of this shortcoming, we still decided to adopt this method because (a) the fish used in this study were too small to burden all sample collections; (b) multiple blood collections on one fish can cause stress, leading to death. Many scientists have attempted to improve the collection method. A new method of the dorsal aorta cannulation technique was developed to take blood samples from individual fish over prolonged periods, thus allowing the advantage of establishing individual PK characteristics (45, 46). Nevertheless, this technique also has limitations as, for example, it reduces appetite and causes a decline in swimming activity, triggering physiological stress due to the cannulation that markedly affects the PK properties (47). Therefore, it is not an ideal method for sample collection.

Additionally, in the current study, oral gavage was used to administer the drug because this method can deliver an exact amount of DC in fish in accordance with their body weight. In future studies, it would be helpful to obtain distinguishable PK results from different dosage forms to enable the easy selection of the optimal drug form. However, natural delivery using medication feed cannot provide an exact dose to individual fish. Moreover, the variability of results is larger and its discrimination is lower than those noted for oral gavage. Accordingly, the properties of natural delivery render it unsuitable for estimating the BA values for selecting the optimal dosage form. However, this treatment approach has been effective in inspecting the depletion of aquatic drug residue in natural settings (48).

This study has some limitations, as blood sampling at each time point was performed using six fish rather than one fish, which did not demonstrate the variability of PKs in fish individuals. The whole DC concentration in plasma was also used to calculate PKPD parameters but not in considering the plasma-protein binding of DC. This is because the only free drug in plasma has antimicrobial activity *in vivo*. Future studies should include further experiments to accurately determine the PKPD parameters.

## Conclusion

This study aimed to determine the BA value of DC and demonstrated its PK properties in channel catfish, following oral and intravenous administration. The results indicated that DC is a good antibiotic in aquatic animals because it presented rapid absorption and distribution, a long half-life but relatively slow elimination due to enterohepatic circulation. Moreover, a medium BA value of 43.84% for DC in channel catfish was calculated. This value is higher than that in tilapia but lower than that in grass carp. These data provide the basis for developing an optimal pharmaceutical formulation in the future. Through these studies, appropriate commercial pharmaceutical preparations could be selected to enhance the therapeutic effect and consequently assist aquatic farmers in reducing economic loss and increasing fish production.

## Data Availability Statement

The raw data supporting the conclusions of this article will be made available by the authors, without undue reservation.

## Ethics Statement

The animal study was reviewed and approved by The Fish Ethics Committee of Yangtze River Fisheries Research Institute, Chinese Academy of Fishery Sciences, Wuhan, China.

## Author Contributions

NX and XA conceived the project. YF, BC, QY, and SZ performed the animal experiment and collected samples. NX calculated PK parameters, contributed to interpretation of data, and drafted the manuscript. YL, JD, and YY analyzed plasma samples by UPLC. XA and YS provided facilities and coordinated the project. All authors read and approved the final manuscript.

## Conflict of Interest

The authors declare that the research was conducted in the absence of any commercial or financial relationships that could be construed as a potential conflict of interest.

## Abbreviations

A: zero–time blood drug concentration intercept of distribution phase
α: distribution rate constant of two-compartmental model
AUC_0*-*144_: area under the concentration (vs.) time curve from 0 to 144 h
AUC_0-∞_: area under the concentration vs. time curve extrapolated to infinity
AUC_0*-*144_/D: area under concentration vs. time curve from 0 to 144 h calibrated by dose
B: zero-time blood drug concentration intercept of elimination phase
β: elimination rate constant of two-compartmental mode
BA: bioavailability
C_max_: the maximum concentration
Cl: systemic total body clearance
DC: doxycycline
K_10_: drug elimination rate constant from central compartment
K_12_: first-order transport rate constant from central compartment to peripheral compartment
K_21_: first-order transport rate constant from peripheral compartment to central compartment
LOD: limit of detection
LOQ: limit of quantification
MIC: minimum inhibitory concentration
NCA: non-compartmental analysis
PK: pharmacokinetic
PKPD: pharmacokinetic-pharmacodynamic
*R*^2^: determination coefficient
RSD: relative standard deviation
t_1/2α_: distribution half-life of two- compartmental mode
t_1/2β_: elimination half-life of two-compartmental model
t_1/2λ*z*_: elimination half-life of non-compartmental model
T_max_: the time to peak concentration
UPLC: ultra-performance liquid chromatography
V_ss_: apparent distribution volume at steady state of two-compartmental model
V_z_: apparent distribution volume at elimination phase of non-compartmental model
λ_z_: elimination rate constant of non-compartmental model.
